# Targeting Immune Dysregulation After Burn Injury for Improved Healing and Outcomes

**DOI:** 10.3390/biom16060806

**Published:** 2026-05-29

**Authors:** Patrick P. G. Mulder, Bouke K. H. L. Boekema, Cornelis H. van der Vlies, Mark W. Fear, Fiona M. Wood, Lucy W. Barrett

**Affiliations:** 1 Burn Research Lab, Alliance of Dutch Burn Care (ADBC), 1941 AJ Beverwijk, The Netherlands; p.p.g.mulder-8@umcutrecht.nl (P.P.G.M.); bboekema@burns.nl (B.K.H.L.B.); 2 Civil-Military Centre of Expertise for Trauma Care (CETC), Central Military Hospital, Defence Healthcare Organisation, Ministry of Defence, 3584 CX Utrecht, The Netherlands; 3 Civil-Military Centre of Expertise for Trauma Care (CETC), Trauma Center, University Medical Centre Utrecht, 3584 CX Utrecht, The Netherlands; 4 Burn Center, Maasstad Hospital, Alliance of Dutch Burn Care (ADBC), 3079 DZ Rotterdam, The Netherlands; vliesc@maasstadziekenhuis.nl; 5 Department of Plastic, Reconstructive and Hand Surgery, Amsterdam UMC, 1105 AZ Amsterdam, The Netherlands; 6 Amsterdam Movement Sciences, Tissue Function and Regeneration, 1081 HV Amsterdam, The Netherlands; 7 Trauma Research Unit, Department of Surgery, University Medical Center Rotterdam (Erasmus MC), 3015 GD Rotterdam, The Netherlands; 8 Department of Trauma and Burn Surgery, Maasstad Hospital, 3079 DZ Rotterdam, The Netherlands; 9 Burn Injury Research Unit, University of Western Australia, Perth, WA 6009, Australialucy.barrett@uwa.edu.au (L.W.B.); 10 Fiona Wood Foundation, Burns Unit, Fiona Stanley Hospital, Murdoch, WA 6150, Australia; 11 Burns Service of Western Australia, Perth Children’s Hospital, WA Department of Health, Nedlands, WA 6009, Australia

**Keywords:** immune response, inflammation, cytokines, surgery, burn injury, immunometabolism

## Abstract

Burn injury induces profound immune dysregulation that extends beyond the acute phase of wound healing, contributing to complications such as delayed repair, infection, and long-term immune dysfunction. Importantly, these effects are not restricted to severe trauma, as similar immune alterations occur following small- to moderate-sized burns. Despite increasing recognition of post-burn immune dysregulation, targeted immunomodulatory therapies remain limited. In this review, we synthesize current insights into the mechanisms driving immune dysfunction after burn injury and outline therapeutic strategies aimed at restoring immune homeostasis. We examine approaches targeting inflammatory triggers and mediators, including acute clinical interventions, reduction in microbial burden, and inhibition of immune cell activation through systemic and local delivery. We also explore strategies to modulate dysregulated innate immune responses by targeting cell-specific functions, such as neutrophil activity and monocyte/macrophage polarization. Persistent activation and exhaustion of the adaptive immune system may be alleviated through interventions such as β-adrenergic blockade, while metabolic, endocrine, and oxidative stress pathways represent additional therapeutic targets. Finally, we highlight key challenges, including the need for improved diagnostics, early prognostic stratification, and personalized treatment approaches to improve outcomes following burn injury.

## 1. Introduction

Burn injury is a severe form of traumatic injury associated with a dysregulated immune response [1,2]. Extensive burns trigger a hyperinflammatory cascade that can lead to systemic inflammatory response syndrome (SIRS) and compensatory anti-inflammatory response syndrome (CARS), which are now recognized as overlapping and interrelated processes that may persist beyond the acute phase of wound healing [3]. This sustained immune imbalance is associated with severe complications, including delayed healing, multiple organ failure, and increased mortality. In addition to damaging acute consequences, many burn survivors develop long-term immune dysfunction. Persistent inflammation, immunosuppression, and catabolism syndrome (PICS), originally described in sepsis and major trauma, reflects a chronic state of immune dysregulation in which concurrent pro- and anti-inflammatory processes drive ongoing immune dysfunction and increased susceptibility to secondary infections [4]. Although the prevalence of PIICS in burn patients remains unclear, early features have been reported in approximately one in six paediatric patients [5]. Notably, while most research has focused on severe burns, growing evidence indicates that even non-severe burn injuries (<20% total body surface area) can induce pronounced acute inflammation and long-term immune alterations [6,7,8,9].

Over recent decades, advances in burn care have markedly improved survival, infection control, wound healing, functional outcomes, and scar quality. However, it is increasingly evident that the impact of burn injury on the immune system extends far beyond the acute phase (reviewed in [10,11]). Burn survivors remain at elevated risk of a wide range of long-term morbidities, including infectious, metabolic, cardiovascular, gastrointestinal, and neuropsychological disorders, as well as malignancy [6]. These observations suggest that burn injury induces persistent systemic alterations, with chronic immune dysfunction emerging as a key underlying contributor to long-term disease risk [1,12]. Despite growing recognition of immune dysregulation following burn injury, there are currently no targeted therapies aimed at restoring immune homeostasis in the treatment arsenal. A major challenge lies in the complexity of the post-burn immune response [13], in which pro- and anti-inflammatory processes are tightly intertwined and essential for effective wound healing, yet may also drive long-term dysfunction if not properly resolved. This creates a narrow and poorly defined therapeutic window for intervention.

In this narrative review, we examine current insights into the immune response to burn injury and explore therapeutic strategies aimed at mitigating immune dysregulation to improve outcomes. Specifically, we discuss key aspects of post-burn immune reactions, with a focus on inflammatory triggers, immune mediators, the innate immune system, the adaptive immune system, and immunometabolic alterations. Finally, we address current challenges and considerations for the development of targeted immunomodulatory interventions. Attention is given to opportunities for drug repurposing and combination therapies, as well as supportive interventions such as changes in nutrition, exercise, and the microbiome, which may contribute to restoring immune balance. Advancing this field will require translating mechanistic insights into targeted, time-sensitive, and patient-specific interventions that restore immune balance and improve acute and long-term clinical outcomes in burn patients.

## 2. Inflammatory Triggers

Burn injuries initiate a multifaceted inflammatory response triggered by both exogenous (non-sterile) and endogenous (sterile) cues [14]. Non-sterile triggers, known as pathogen-associated molecular patterns (PAMPs), originate from microorganisms entering the disrupted skin and include lipopolysaccharide (LPS), lipoteichoic acid (LTA), flagellin, and foreign nucleic acids [15,16]. In parallel, thermal injury causes extensive tissue damage, resulting in the release of sterile triggers collectively referred to as damage-associated molecular patterns (DAMPs), which are derived from necrotic cells, denatured proteins, and disrupted extracellular matrix components. Together, these signals activate an immune response and shape the early inflammatory cascade (Table 1).

**Figure 1:**
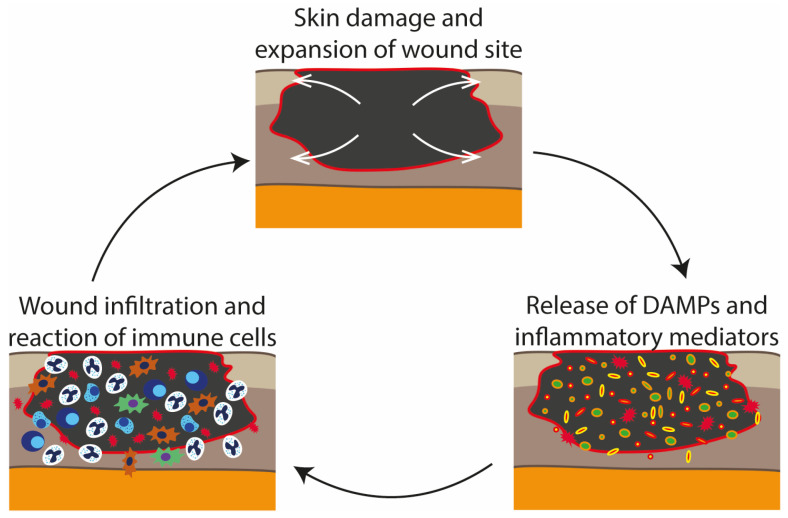
Vicious cycle of inflammation and tissue damage that can be established after burn injury. The three displayed skin layers from upper to lower represent the epidermis (light grey), dermis (dark grey) and hypodermis (orange). The black area with a red border represents the burned skin tissue (eschar). The upper figure shows expansion of the wound area (white arrows); the lower right figure shows the release of DAMPS and inflammatory mediators; the lower left figure shows infiltration of various immune cells.

PAMPs and DAMPs are sensed by pattern recognition receptors (PRRs) expressed not only on immune cells, but also on keratinocytes, fibroblasts, and endothelial cells [16]. Upon ligand binding, PRRs initiate intracellular signalling pathways, including NF-κB, MAPKs, and inflammasomes, that promote cytokine production and immune cell activation [17,18]. While concentrations of these danger signals are particularly high within the burn wound microenvironment, patients with extensive burns often experience systemic dissemination of inflammatory triggers, resulting in sustained PRR signalling in circulating leukocytes [19,20]. Persistent exposure to inflammatory triggers can drive a paradoxical immune phenotype characterized by cellular activation accompanied by functional impairment, sometimes described as immune exhaustion or senescence [21,22,23,24]. Importantly, the burn wound itself remains a continuous source of inflammatory stimuli. Ongoing tissue necrosis, microbial colonization, and expansion of the injured area promote further release of PAMPs and DAMPs, establishing a self-reinforcing cycle in which inflammation promotes further tissue damage and the generation of additional danger signals. This process perpetuates systemic immune activation and ongoing tissue injury (Figure 1) [25]. In addition, complement activation acts as an amplification loop linking DAMP signalling to downstream inflammatory and cellular responses [26]. In severe cases, these inflammatory signals can become overwhelming and trigger SIRS, characterized by excessive cytokine production and widespread activation of innate immune cells, leading to systemic inflammation, organ dysfunction, and even death. Collectively, this dynamic process transforms the burn wound into a persistent driver of immune dysregulation. Targeting inflammatory triggers, therefore, represents a key therapeutic strategy for improving outcomes in burn care.

### 2.1. Strategy 1: Control of Bacterial Colonization

Disruption of the skin barrier following burn injury allows microorganisms to enter and colonize the wound. The burn eschar provides a nutrient-rich environment that facilitates microbial growth, enabling bacteria to readily colonize necrotic tissue [27]. Controlling bacterial colonization, therefore, remains a cornerstone of burn care to prevent local infection, systemic complications, and excessive inflammatory signalling [1]. Early assessment of microbial burden through wound cultures and diagnostic testing is essential for guiding antimicrobial therapy and enabling timely intervention [28]. Antibiotics are not usually indicated prophylactically, even in selected high-risk patients, but rather preferentially used as targeted therapy once pathogens and their sensitivity are identified. Selective digestive decontamination aims to suppress potentially pathogenic microorganisms from the oropharynx and gastrointestinal tract while preserving anaerobic flora, thereby reducing the risk of translocation and subsequent systemic infection. This approach has been explored in burn patients [29]. However, a more commonly used strategy is the use of nutrition to maintain normal gut function wherever possible. Thus, controlling endogenous sources of infection may indirectly attenuate systemic inflammatory responses following severe burn injury.

Topical antimicrobial dressings are widely used to reduce bacterial load within the wound environment [30]. Silver-based formulations, such as silver sulfadiazine or nanocrystalline silver dressings, remain commonly applied for their broad antimicrobial activity, while agents such as mafenide acetate or mupirocin may provide additional coverage against specific pathogens including Pseudomonas aeruginosa or Staphylococcus aureus. Effective control of bacterial colonization not only limits infection risk but also reduces the burden of PAMPs, thereby decreasing inflammation triggered by microbial components. However, antimicrobial strategies alone are often insufficient because necrotic burn tissue continues to act as a reservoir for both microbes and inflammatory mediators. Consequently, removal of devitalized tissue remains a critical strategy in infection control.

### 2.2. Strategy 2: Removal of Burn Tissue (Eschar)

Removal of burn wound eschar (dead, black/brown, leathery tissue areas) is central to limiting both bacterial colonization and sterile inflammation. Necrotic tissue perpetuates the inflammatory cascade not only by serving as a niche for microbial growth but also as a major source of DAMPs. Early excision of eschar has been shown to reduce systemic cytokine levels, hypermetabolism, and sepsis risk, thereby improving clinical outcomes [31]. The immunomodulatory effect of debridement extends beyond infection control. By removing necrotic tissue, debridement reduces the continuous release of DAMPs originating from dying cells and degraded extracellular matrix components, thereby limiting sustained immune activation.

Eschar removal can be achieved through several approaches. Surgical excision performed using a scalpel, Weck blade, dermabrasion, or hydrosurgery remains the gold standard in many burn centers. Early excision, typically performed within the first week after injury, is associated with shorter hospital stays, reduced systemic inflammation, and improved wound healing [28]. Enzymatic debridement, such as bromelain-based formulations (e.g., Nexobrid^®^), offers a selective alternative approach that removes denatured and necrotic tissue while preserving viable dermis, which may accelerate re-epithelialization and improve functional outcomes [32]. After the eschar is excised, expedient restoration of the skin integrity is essential to establish a functional barrier, to mitigate against ongoing inflammation, fluid losses and the risk of secondary infection.

In several European settings, including the Netherlands, silver sulfadiazine–cerium nitrate dressings (Flammacerium^®^) are used not only for their antimicrobial properties but also for their ability to modulate the wound environment. Preclinical and clinical observations suggest that such dressings can limit burn wound infection while attenuating inflammatory responses within the eschar [33]. Together, these approaches highlight that effective eschar management extends beyond tissue removal alone and may also involve stabilization and modulation of the wound microenvironment. By reducing both microbial burden and the release of inflammatory triggers, these strategies represent a critical step in interrupting the cycle of burn-induced immune activation.

### 2.3. Strategy 3: Blocking Inflammatory Triggers

Targeting upstream danger signals themselves represents an attractive strategy to attenuate inflammation before PRR activation and downstream cytokine amplification occur. In burns, several DAMPs, including high mobility group box 1 (HMGB1), S100 proteins, extracellular DNA, ATP, and uric acid, have been implicated in systemic inflammation and immune dysregulation [34,35]. HMGB1 is one of the most extensively studied DAMPs in trauma and sepsis. Neutralization of HMGB1 using monoclonal antibodies has been shown to reduce systemic cytokine release and organ injury in experimental sepsis models [36]. Because circulating HMGB1 levels remain elevated after severe burns, similar therapeutic strategies may hold translational potential in burn-induced hyper-inflammation [34]. Small-molecule inhibitors such as glycyrrhizin, which directly bind HMGB1 and inhibit its inflammatory activity, have demonstrated beneficial effects in several inflammatory disease models [37,38] and may represent candidates for drug repurposing. and may represent candidates for drug repurposing. Blocking complement factors offers another opportunity to interfere with inflammatory triggers. Excessive generation of anaphylatoxins such as C3a and C5a contributes to neutrophil recruitment, vascular permeability, and sustained inflammation, while also promoting immune dysfunction at later stages [26]. Therapeutically targeted inhibition of complement components, such as blockade of C5 or C5a-C5aR signalling using eculizumab or vilobelimab, has shown beneficial effects in other inflammatory diseases and may attenuate tissue injury and organ dysfunction [39,40].

Extracellular nucleic acids represent another important category of inflammatory triggers. Circulating cell-free DNA and extracellular traps (ETs) contribute to persistent inflammation and microvascular injury following burn trauma [24]. Enzymatic degradation of extracellular DNA using recombinant DNase I (dornase alfa), a therapy widely used in cystic fibrosis, has demonstrated anti-inflammatory effects in experimental models of sepsis and acute lung injury, where it reduces neutrophil ET burden and improves tissue function [41]. While these conditions share features with severe burns regarding inflammatory reactions, the efficacy of such therapeutic strategies in burn patients is yet to be investigated. Translation to burn care requires consideration of key challenges, including optimal timing of intervention, route of administration (systemic versus topical delivery to the wound), and the risk of impairing antimicrobial defence, as ETs also contribute to pathogen clearance. Additional strategies include enzymatic degradation of extracellular ATP or pharmacological inhibition of purinergic signalling pathways that activate inflammasomes [42]. By neutralizing or removing molecular danger signals, such approaches aim to reduce the inflammatory load at its source and thereby limit both hyperinflammation and the subsequent development of immune exhaustion. Although these approaches offer a promising means to reduce upstream inflammatory triggers, their clinical application in burn patients will depend on achieving a balance between dampening harmful inflammation and preserving essential host defence mechanisms.

### 2.4. Strategy 4: Blocking Activation

An alternative therapeutic strategy involves targeting the receptors and intracellular signalling pathways that translate PAMP and DAMP recognition into inflammatory gene expression and protein production. Toll-like receptor (TLR) signalling plays a central role in burn-induced inflammation [43]. Pharmacological inhibition of TLR4 using antagonists such as eritoran has been shown to reduce cytokine production and tissue injury in preclinical models; however, human clinical trials in sepsis did not demonstrate a survival benefit [44]. These studies provide proof-of-concept for targeting TLR signalling, which may warrant re-evaluation in severe burns. Decoy receptor strategies offer a complementary approach. Soluble receptor for advanced glycation end products (sRAGE) can sequester ligands such as HMGB1 and S100 proteins, thereby limiting RAGE-mediated inflammatory signalling [45]. In models of sepsis and acute lung injury, sRAGE reduces cytokine release and tissue damage [46], supporting its potential to attenuate sterile inflammation. At the level of cytosolic PRRs, inhibition of inflammasome activation has emerged as a promising strategy. The NLRP3 inflammasome, activated by burn-associated DAMPs such as ATP and uric acid, can be selectively inhibited by small molecules such as MCC950, which has demonstrated efficacy across multiple inflammatory disease models [47]. Because oxidative stress amplifies DAMP signalling [48], antioxidant therapies may provide an important adjunct. Modulation of signalling pathways, currently explored in cancer and sepsis, may help restore immune balance after burn injury. Collectively, these approaches aim not to suppress innate immunity, but to recalibrate inflammatory signalling and restore effective immune function.

Burn injury induces a sustained release of PAMPs and DAMPs that drive systemic immune activation through PRR and inflammasome signalling pathways, contributing to subsequent immune dysfunction. Therapeutic strategies can therefore target multiple levels of this cascade, including reducing the source of inflammatory stimuli through infection control and debridement, neutralizing circulating danger signals, and modulating PRR- and inflammasome-mediated signalling. Emerging approaches, including DAMP-targeting therapies, receptor antagonists, and antioxidant strategies, highlight the potential of selectively recalibrating rather than suppressing innate immune responses.

## 3. Immune Mediators

Inflammatory mediators, which include cytokines, chemokines, and growth factors, are small signalling proteins that facilitate cell-to-cell communication and play a critical role in wound healing [49]. Cytokines, a broad category of secreted proteins, are chemical messengers that modulate the immune system, both during homeostasis and in response to injury. Chemokines are small chemotactic cytokines specialising in cell movement, recruiting cells to the site of injury and regulating angiogenesis [50], while growth factors direct cellular proliferation and differentiation. Effective wound healing requires precise and coordinated expression of these mediators, which shift dynamically across the different phases of skin repair. Imbalances or aberrant expression of these mediators after injury are associated with poor outcomes, including delayed wound closure and pathological scarring [51] (3). A clearer understanding of the exact roles, both protective and pathogenic, will be essential to determine whether targeting inflammatory mediators represents a viable therapeutic strategy for restoring immune function in burn survivors. Inflammatory mediators involved in the acute and long-term response to burn injury are shown in Table 2.

The complexity of the immune response to burn injury, especially following severe burns (classified as affecting >20% of the total body surface area, TBSA), has been extensively reviewed [1,3,13,52,53]. Compared to other forms of trauma, burns induce a more intense and prolonged inflammatory response that differs in both the timing and profile of immune mediators produced across all stages of wound healing [1,54]. The acute phase of burn injury is characterised by a rapid increase in the level of systemic inflammatory mediators to navigate immune cells towards the injury site [11,55,56]. In severe burns, elevated early serum levels of interleukin (IL)-6, IL-8, G-CSF and MCP-1 have been associated with an increased risk of mortality [57]. Concurrently, inflammatory mediators rapidly infiltrate burn wound tissue, with pro-inflammatory mediators found to be particularly elevated in burn eschar [11,58].

While the acute systemic response to burn injury is well characterised, the persistence of aberrant inflammatory signalling, especially in patients with non-severe burns (classified as affected <20% TBSA), has only recently been described. Early studies in severely burned children demonstrated sustained elevation of GM-CSF, IFN-γ, TNF-α, IL-1β, IL-2, IL-5, IL-7, IL-10, and IL-17 for up to three years post-injury [55]. More recent studies have extended these findings to non-severe burns, with paediatric patients exhibiting elevated circulating IL-2, IL-7, TNF-α, and IFN-γ up to three years after injury compared to age-matched controls [8]. A 2024 study further reported increased levels of IL-17, TNF-α, NFκβ, and CCR6 at 18 months post-injury in a similar cohort [7]. Together, these findings highlight the potential for long-term immune dysregulation following burn injury, irrespective of burn severity, and underscore the persistence of both pro- and anti-inflammatory signalling abnormalities. Considering the significant role inflammatory mediators play in the response to burn injury and their involvement in complications, treatments to counteract excessive aberrant signalling have been explored, including treatments to block or induce the expression of specific mediators, alongside broader anti-inflammatory treatments.

**Table 2:** Inflammatory mediators active in the immune response to burn injury.

Inflammatory Mediators	Mediator	Pro/Anti-Inflamm.	Local (Acute Burn Wound)	Systemic (Acute)	Systemic (Chronic)	Cells Involved (Secretors and Affected Cells)	Role in Wound Healing (Phase)
**Cytokines**	IL-1α	Pro	Decreased [11]	No change	U	Source: K, Mac, Epi Role: Proliferation & differentiation of fibroblasts; keratinocyte activation	Inflammatory Proliferation
	IL-1β	Pro	Increased [11,58,59,60]	Increased [60]	Increased [55]	Source: N, Mac, Mo, Endo, Epi, injured K. Role: M1 Macrophage differentiation; T cell recruitment	Inflammatory
	IL-2		Increased [11]	Increased [60]	Increased [8,55]	Source: T Role: T cell proliferation. Amplifies adaptive immune cell activation in burns	Inflammatory
	IL-4	Anti	U	Increased [60]	U	Source: T, Mast, E Role: M2 macrophage polarisation; promotes tissue repair and fibrosis	Proliferation Remodelling
	IL-5	Anti	U	Increased [60]	Increased [55]	Source: T, Mast Role: Eosinophil activation	Inflammatory
	IL-6	Pro	Increased [11,59]	Increased [12,55,56,60,61]	U	Source: Mac, N, T, B Mo, Endo, Epi. Role: M1 Macrophage differentiation; systemic inflammation. SIRS	Inflammatory
	IL-7	Dual	U	Increased [60]	Increased [8,55]	Source: F, K Role: T cell survival and homeostasis.	Resolution
	IL-10	Anti	U	Increased [55,56]	Increased [8,55]	Source: T, Mo, B, Mac Role: Suppresses inflammation; promotes resolution	Resolution Remodelling
	IL-12p70	Pro	U	Increased [60]	U	Source: DC, Mac Role: Drives Th1 differentiation	Inflammatory
	IL-13	Anti	U	Decreased [56,60]	Decreased [56]	Source: Th2 Role: M2 macrophage polarisation; fibrosis scar formation	Proliferation Remodelling
	IL-15	Pro	U	Increased [61]	U	Source: Mo, DC, K Role: NK & T cell activation	Inflammatory
	IL-17	Pro	U	U	Increased [55]	Source: T Role: Neutrophil recruitment; pro-keratinocyte	Inflammatory
	IL-18	Pro	Decreased [11]	U	U	Source: K, Mac Role: Promotes Th1 responses	Inflammatory
	IL-33	Dual	Decreased [11]	U	U	Source: K, Endo, F Role: Activates innate immune cells; alarmin	Inflammatory
	IFN-γ	Dual	Increased [11]	U	Increased [8,55]	Source: Th1, NK Role: Inflammatory drivers	Inflammatory
	TNF-α	Pro	Increased [11,59]	U	Increased [7,8,55]	Source: N, T, Mac, B, Mo, NK, Endo, Epi, Adi. Role: Inflammatory drivers; increases vascular permeability and recruits immune cells	Inflammatory
**Chemokines**	MCP-1 (CCL2)	Pro	Increased [11,58]	Increased [55,56,61]	U	Source: Mac, F, Endo, K Role: Macrophage/neutrophil recruitment; promotes M1 -> M2 macrophage transition	Inflammatory Proliferation
	MIP-1α (CCL3)	Pro	Increased [11]	Increased [61]	U	Source: Mac, N Role: Macrophage and lymphocyte recruitment; NK activation	Inflammatory
	MIP-1β (CCL4)	Pro	Increased [11]	U	U	Source: Mac, DC Role: Macrophage & lymphocyte recruitment	Inflammatory
	RANTES (CCL5)	Pro	Increased [11]	U	U	Source: T, Pl, Endo Role: Macrophage & T cell recruitment	Inflammatory
	IL-8 (CXCL8)	Pro	Increased [11,58]	Increased [56,61]	U	Source: K, Mac, Endo Role: Neutrophil recruitment & activation	Inflammatory
	SDF-1 (CXCL12)	Dual	Increased [62]	U	U	Source: F, Endo Role: Stem cell recruitment; angiogenesis	Proliferation Angiogenesis
	MIP-3α (CCL20)	Pro	Decreased [11]	U	U	Source: K Role: Immune cell recruitment	Inflammatory
	GROα	Pro	Increased [11,58]	U	U	Source: K, Mac, Endo Role: Neutrophil & immune cell recruitment	Inflammatory
	IP-10 (CXCL10)	Pro	Increased [11]	U	U	Source: K, Mac, Endo Role: T cell recruitment; immune regulation	Inflammatory Proliferation
	CTACK (CCL27)	Dual	Decreased [11]	U	U	Source: K Role: Skin-specific T cell recruitment	Inflammatory
**Growth factors**	VEGF-A	Pro	Increased [11,59,62,63,64]	Increased [61]	U	Source: M2 Mac, K, F Role: M2 Macrophage differentiation; fibroblast & endothelial regulation	Proliferation Angiogenesis
	TGF-β1	Dual	Increased [11,59,63]	U	U	Source: F, K, Mac, Pl. Role: Fibroblast regulation, M2 Macrophage differentiation and recruitment; scar formation & ECM deposition; pro-fibrotic	Proliferation Remodeling
	TGF-β2	Dual	Decreased [11]	U	U	Source: F, K Role: Regulates ECM deposition & cell proliferation; pro-fibrotic	Proliferation Remodelling
	TGF-β3	Anti	Increased [59]	U	U	Source: F, K Role: Fibroblast regulation; anti-fibrotic	Remodelling
	PDGF-AA	Pro	Decreased [11]	U	U	Source: Pl, MacRecruit and activate fibroblasts and endothelial cells	Angiogenesis Proliferation Remodelling
	PDGF-BB	Pro	Decreased [11]	U	U	Source: Pl, Mac Role: Recruit and activate fibroblasts & endothelial cells	Angiogenesis Proliferation Remodelling
	G-CSF	Pro		Increased [56,61]	U	Source: Mac, Endo, F Role: Neutrophil recruitment; inflammatory	Inflammatory
	GM-CSF	Pro	Decreased [11]	Increased [61]	Increased [55]	Source: T, Mac, F, Endo Role: Mono/Mac activation	Inflammatory
	EGF	Repair	U	Increased [56]	U	Source: Keratinocytes Role: Proliferation & differentiation of epithelial cells; keratinocytes & fibroblasts	Proliferation
	FGF	Repair	Increased [63]	U	U	Source: K, F, Epi Role: Proliferation of fibroblasts & keratinocytes; keratinocyte migration; fibroblast regulation	Angiogenesis Proliferation
**Transcription factors**	HIF-1α	Dual	Increased [62]	U	U	Source: K, Mac, Endo Role: Hypoxia response; angiogenesis	Proliferation
	NFκβ	Pro	U	Increased [7]	U	Source: Mac, K, Endo Role: Master regulator of inflammatory gene expression	Inflammatory

Abbreviations: U = unknown (not investigated), Interleukin (IL-), Interferon gamma (IFN-γ), Tumour-necrosis factor alpha (TNF-α), Chemokine C-X-C motif ligand (CXCL_), Monocyte chemo-attractant protein (MCP-), Monocyte inducible protein (MIP-), Regulated on Activation, Normal T Cell Expressed and Secreted (RANTES), Stromal cell-derived factor 1 (SDF-1), Growth-regulated oncogene alpha (GROα), CC chemokine ligands (CCL_), Interferon-gamma-inducible protein 10 kDa (IP-10), Cutaneous T-cell-attracting chemokine (CTACK), Vascular endothelial growth factor (VEGF-A), Transforming growth factor (TGF-), Platelet-derived growth factor (PDGF-), Granulocyte colony-stimulating factor (G-CSF), Granulocyte-macrophage colony-stimulating factor (GM-CSF), Epidermal growth factor (EGF), Fibroblast growth factor (FGF), Hypoxia-inducible factor 1α (HIF-1α), Nuclear factor kappa-light-chain-enhancer of activated B cells (NF-κB), T: T cells, B: B cells, Mo: Monocytes, Mac: Macrophages, N: Neutrophils, NK: Natural killer cells, K: Keratinocytes, F: Fibroblasts, Epi: Epithelial cells, Pl: Platelets, Endo: endothelial cells, Adi: Adipose cells.

### 3.1. Strategy 1: Systemic Targeting of Inflammatory Mediators

IL-6 is a key driver of acute hyperinflammatory responses following burn injury, and therapeutic blockade of excessive IL-6 signalling has been explored during the acute phase. In a mouse model of severe burn injury, systemic administration of an anti-IL-6 monoclonal antibody reduced post-burn hypermetabolism, including cachexia, adipose tissue browning, and liver toxicity [63]. Improvements in skin pathology were also observed, potentially through downstream modulation of TGF-β1 and VEGF signalling pathways associated with aberrant scarring. Clinical evidence in burns remains limited; however, IL-6 receptor blockade with tocilizumab has been reported in a single paediatric case of severe burn (91% TBSA), where it was used to successfully manage cytokine storm [65]. Despite these promising findings, tocilizumab is associated with an increased risk of infection, restricting its use in burn care to severe or refractory cases [66].

Other inflammatory mediators that represent potential therapeutic targets include IL-1β and TNF-α, which remain chronically elevated following burn injury. IL-1β has recently been implicated in post-burn immune dysfunction through the formation of complexes with HMGB1 that are enriched in tissue-resident and circulating plasma microvesicles [67]. Canakinumab, a monoclonal antibody targeting IL-1β, is approved for use in a number of autoinflammatory diseases [68] and has been shown to reduce systemic IL-6 levels [69]; however, it has not yet been investigated in the context of burns. Treatments targeting TNF-α, a key regulator of chronic inflammatory pathways, are also widely used in inflammatory disorders such as rheumatoid arthritis, psoriatic arthritis, and ulcerative colitis (reviewed in [70]). In these settings, TNF-α inhibition can restore immune homeostasis by reducing downstream pro-inflammatory cytokines, including IL-1, IL-6, and IFN-γ. Although not yet evaluated clinically in burn patients, preclinical studies in rodent models demonstrate that systemic TNF-α inhibition can mitigate burn-associated cardiac and bone dysfunction [71,72]. However, TNF-α inhibitors are associated with significant adverse effects in other disease contexts, including neurological [73] and dermatological [74] complications, highlighting the need for caution when considering cytokine-targeted therapies in burns.

A major limitation of systemic therapies targeting individual inflammatory mediators is their potential to induce immunosuppression, increasing susceptibility to bacterial, fungal, and viral infections, which is of particular concern for burn patients [75]. In addition, inflammatory mediators play essential roles in wound healing, limiting the feasibility of broadly suppressing these pathways. As such, cytokine-targeted therapies are likely to be restricted to severe or refractory cases. Differences in efficacy and safety between adult and paediatric populations further emphasise the need for improved mechanistic understanding and patient stratification [76].

### 3.2. Strategy 2: Local Targeting of Inflammatory Factors

Given the risks associated with systemic therapies, local delivery of therapeutics targeting inflammatory mediators has been explored for the treatment of burn wounds. These include cytokine inhibitors conjugated to biomaterials such as hyaluronic acid, as well as incorporation into hydrogels or advanced wound dressings for site-specific delivery. In a mouse model of skin transplantation, local anti-IL-6 treatment administered via a GelMA hydrogel enhanced the survival of skin allografts with superior efficiency to systemic anti-IL-6 administration [77]. Similarly, in a rat model of partial-thickness burn injury, local delivery of an anti-TNF-α monoclonal antibody conjugated to hyaluronic acid reduced burn wound progression and local inflammation [78]. In contrast, anti-IL-6 treatment in the same model did not confer significant benefit, highlighting variability in the effectiveness of targeting individual cytokines in different contexts.

Local delivery of growth factors has also been investigated to enhance wound healing and angiogenesis. Administration of recombinant epidermal growth factor (EGF) accelerates burn wound healing in a mouse model of burn injury [79]; however, high levels of EGF are associated with pathological scarring [80], reflecting the complex and context-dependent roles of growth factors in tissue repair. Despite demonstrated benefits for other injuries (reviewed in [81]), topical application of growth factor to enhance burn wound healing has not been clinically tested, and the involvement of growth factors including TGF-β and VEGF in tumour formation indicates significant research will be required to determine the relevance of growth factor therapies in burn wound healing and how growth factor-based therapeutics might impact long-term outcomes.

### 3.3. Strategy 3: Broad Anti-Inflammatory Therapies

Glucocorticoid steroids (GCs) are widely used anti-inflammatory drugs that mimic cortisol, a stress hormone significantly upregulated in response to burns [82]. Dexamethasone, a potent long-acting corticosteroid, exerts broad immunosuppressive effects, including upregulation of IL-10 and suppression of pro-inflammatory cytokines such as IL-1 and IL-6 (reviewed in [83]). Given their effects on both innate and adaptive immune responses, GCs have been considered for use in burn treatment. However, preclinical studies indicate that dexamethasone may have unintended consequences in this context. Similar to cytokine-targeting strategies, GC use is associated with an increased risk of infection, limiting its applicability. Notably, a recent study using dexamethasone-eluting endotracheal tubes in a murine model of burn-related inhalation injury reported increased biofilm formation and elevated cytokine levels, suggesting that dexamethasone may be unsuitable during the acute phase of burn injury [84].

Colchicine is a broad anti-inflammatory drug that has been used for decades to treat inflammatory disorders, including some skin diseases [85]. More recently, it has been shown to reduce cardiovascular risk, partly through inhibition of IL-1β signalling [86]. Mechanistically, colchicine exerts anti-inflammatory effects by inhibiting neutrophil recruitment and activation, reducing circulating levels of IL-1β, IFN-γ, IL-18, and IL-6, and suppressing TGF-β1-mediated fibrotic pathways (reviewed in [87]). In addition, colchicine influences immunometabolic processes, including reductions in reactive oxygen species and nitric oxide production. While these properties suggest potential relevance in burn-associated inflammation, their application in this setting remains unexplored, and further studies are required to determine their safety and efficacy in the context of post-burn immune dysfunction.

## 4. Innate Immune System

The innate immune system provides the first line of cellular defence following burn injury, mounting a rapid, non-specific response that is essential for pathogen clearance and tissue repair. Innate immune cells, including neutrophils, monocytes/macrophages, dendritic cells, and other innate lymphoid populations, are rapidly recruited to the wound site and activated via PRRs [19,43]. However, this response is often dysregulated in severe burns, with excessive or prolonged activation contributing to tissue damage, vascular leakage, and systemic inflammation, while concurrent functional impairment increases cell exhaustion and overall susceptibility to infection. Neutrophils, as early responders, play a key role in antimicrobial defence through phagocytosis, reactive oxygen species (ROS) production, and neutrophil extracellular trap (NET) formation, but can also drive collateral tissue injury. Similarly, monocytes and macrophages are essential for debris clearance and coordination of repair, yet burn injury can skew their polarization toward a sustained pro-inflammatory (M1) phenotype, instead of a pro-healing (M2) phenotype. Dendritic cell dysfunction post-burn further compromises antigen presentation and thereby adaptive immune activation.

### 4.1. Strategy 1: Modulation of Neutrophil Responses

Neutrophils are rapidly recruited to burn wounds and play a central role in early antimicrobial defence; however, excessive or prolonged activation contributes to collateral tissue damage, immune dysfunction, and impaired wound healing. Neutrophil dysfunction, increased immature granulocyte counts, and elevated circulating cell-free DNA have been proposed as biomarkers for early detection of sepsis following burn injury, and may actively contribute to its pathogenesis [12,24,88]. Consequently, limiting excessive neutrophil recruitment represents an important therapeutic strategy. Key mediators driving neutrophil recruitment and activation include complement factor C5a, IL-8/CXCL8, MCP-1/CCL2, and G-CSF. Pharmacological inhibition of these pathways, including blockade of C5a/C5aR1 or CXCR1/2, has been shown to reduce neutrophil infiltration and tissue injury in models of sepsis and acute lung injury, and may hold translational potential in burn injury [89,90,91].

In addition to limiting recruitment, targeting neutrophil-mediated tissue damage is a key strategy. Activated neutrophils release ROS, proteolytic enzymes such as neutrophil elastase and myeloperoxidase (MPO), and form NETs, all of which contribute to endothelial damage and impaired tissue repair [92]. Inhibition of NET formation, for example, using peptidylarginine deiminase 4 (PAD4) inhibitors or enzymatic degradation of extracellular DNA using DNase I, has demonstrated anti-inflammatory effects in models of sepsis and sterile injury [41]. Similarly, neutrophil elastase inhibitors such as sivelestat can reduce tissue damage and have shown clinical benefit in inflammatory conditions, including acute lung injury and COVID-19 [93], while MPO inhibition may further attenuate oxidative injury [94]. Targeting oxidative stress through antioxidant therapies, such as N-acetylcysteine, represents an additional approach to reduce ROS-mediated damage while preserving immune function [95]. Importantly, therapeutic strategies should aim to steer rather than completely suppress neutrophil responses, as preservation of antimicrobial activity remains critical in burn patients [93].

### 4.2. Strategy 2: Targeting Monocyte and Macrophage Polarization

Monocytes and macrophages are highly plastic innate immune cells that coordinate both inflammation and tissue repair following burn injury. Recruited monocytes differentiate into macrophages that adopt pro-inflammatory (M1) or pro-repair (M2) phenotypes. In burn patients, this balance is frequently disrupted, with sustained M1 polarization and impaired transition toward M2 contributing to persistent inflammation, tissue damage, and delayed wound healing [96,97,98]. Interventions could therefore aim to restore balanced macrophage responses for improved healing and resolution. Cytokine-based approaches, including IL-4 or IL-10, promote M2 polarization and enhance tissue repair in experimental models [99]. In addition, biomaterial-based strategies, such as engineered scaffolds or advanced wound dressings, can locally modulate macrophage behaviour within the wound microenvironment to support regeneration. Mouse studies demonstrated that lncRNA X-inactive-specific transcript, which targets IL-33, can promote burn wound healing through M2 macrophage activation [100]. Clinically, recombinant GM-CSF has shown efficacy in accelerating burn wound healing, likely in part through enhancement of monocyte/macrophage function [101,102].

Beyond polarization, burn injury is also associated with functional impairment of circulating monocytes, including reduced HLA-DR expression and diminished cytokine production, reflecting a state of immune suppression [34,103]. At the same time, activation of inflammatory pathways such as the NLRP3 inflammasome contributes to early amplification of inflammation while predisposing to later immune dysfunction [18,35]. These findings highlight the dual challenge of limiting excessive inflammation while restoring effective immune function. Importantly, therapeutic modulation of monocytes and macrophages should be temporally controlled. Early inflammatory responses are critical for pathogen clearance, whereas later phases require resolution and tissue repair. Accordingly, interventions should aim to fine-tune macrophage responses over time, promoting an appropriate transition from pro-inflammatory to pro-repair phenotypes without compromising host defence.

### 4.3. Strategy 3: Enhancing Antigen Presentation and Innate Immunity

Beyond neutrophils and macrophages, additional innate immune populations play critical roles in coordinating host defence and bridging adaptive immunity after burn injury. Dendritic cells (DCs) are essential for antigen presentation and T cell activation. In a rat model of severe burns, both the number and functional capacity of circulating and tissue-resident DCs were found to be reduced, leading to impaired antigen presentation and weakened adaptive immune responses [104]. This dysfunction contributes to post-burn immunosuppression and increased susceptibility to secondary infections. Therapeutic strategies aimed at restoring DC function, therefore, represent a promising avenue to enhance immune competence. Other innate immune cells, including natural killer (NK) cells and mast cells, further contribute to immune regulation following burn injury. Mast cells are rapidly activated following tissue injury and release histamine, cytokines, and proteases that regulate vascular permeability, immune cell recruitment, angiogenesis and tissue remodelling. While these responses are essential for early host defence, mouse studies have demonstrated excessive mast cell activation may exacerbate inflammation and tissue damage [105,106], and persistent activation has been associated with enhanced fibrotic responses and scar formation in cutaneous wound models [107]. Although mast cells, as well as NK cells and innate lymphoid cells, are less extensively studied in burn injury compared to neutrophils and macrophages, they might play important roles in regulating the balance between immune activation and suppression, warranting further investigation in humans. Targeting antigen presentation and innate immune competence may therefore complement other immunomodulatory strategies by restoring effective host defence, while avoiding excessive inflammation.

Excessive activation of neutrophils and pro-inflammatory macrophages after burn injury contributes to tissue damage and sustained inflammation, while dysfunction of monocytes and dendritic cells impairs immune competence and increases susceptibility to infection. Therapeutic strategies should therefore aim to rebalance, rather than suppress, innate immune responses by limiting harmful inflammation, promoting resolution and repair, and restoring effective antigen presentation. Achieving this requires careful temporal and patient-specific modulation, with combination approaches targeting multiple innate pathways likely offering the greatest potential to restore immune homeostasis following burn injury.

## 5. Adaptive Immune System

The adaptive immune system consists of professional immune cells, including T and B cells, that act through specialised receptors to recognise, respond to, and remember specific antigens to provide superior protection against new and returning pathogens [107]. Rapid and precise regulation of adaptive immune cells is essential for both immune homeostasis and effective responses to immune challenges. This regulation is also inextricably linked to cellular metabolism, as adaptive immune cells must dynamically adjust their energy demands during activation, proliferation, and differentiation. The influence of metabolic pathways on immune cell fate and function, collectively termed immunometabolism, is increasingly being recognised as a key factor in disease pathogenesis. Persistent hypermetabolism is a hallmark of severe burns [108], and emerging data demonstrates that even smaller burns can cause long-term metabolic reprogramming [9,109]. Given the central role of immunometabolism in shaping adaptive immune responses, this section will outline the current understanding of adaptive immune responses to burn injury and associated metabolic processes and consider how therapeutic targeting of immunometabolic pathways may help mitigate chronic immune dysfunction after burns.

Burn injury is associated with delayed, excessive activation of adaptive immune responses, followed by an accumulation of exhausted and dysfunctional T cells in circulation that can persist long after wound healing [6,13,52]. In normal wound healing, T regulatory cells (Tregs) act to suppress inflammatory processes to reduce tissue damage, while skin resident Tregs are actively involved in remodelling and epithelial barrier repair [110]. Effective wound closure also depends on coordinated antigen presentation between innate and adaptive immune cells [111,112], with aberrant T cell activity implicated in fibrosis and scarring [113,114,115]. Studies in mice demonstrate burn injury triggers a rapid increase in pro-regenerative skin resident Gamma-Delta (γδ) T cells, which recruit other immune cells, including Alpha-Beta (αß) T cells, to the skin [116]. However, these αβ T cells often exhibit altered activation states, including a CD4^−^CD8^−^ phenotype and aberrant expression of activation markers, consistent with suppression of conventional αβ T cell function alongside enhanced γδ T cell activity in the early wound environment. In humans, increased infiltration of B cells and pro-inflammatory γδ T cells has similarly been observed in burn eschar during the weeks following injury [11]. Collectively, these findings demonstrate the complex and dynamic role of adaptive immune cells in wound healing, which becomes dysregulated immediately following burn injury and may contribute to both impaired immune function and pathological tissue repair.

After a prolonged acute response to burn, circulating T cells remain chronically activated and functionally impaired. A longitudinal study in paediatric burn survivors over 18 months identified a persistent increase in circulating natural killer T cells, activated γδ T cells expressing high levels of pro-inflammatory mediators IL-17 and NFκB, and aberrant inflammatory skin-homing T regulatory cells (CCR4^+^ CCR6^+^ Tregs) characterised by reduced IL-10 and elevated TNFα [7]. These populations increased over time, indicating a sustained shift in adaptive immunity towards chronically activated, highly specialised T cell subsets, despite complete wound healing. Chronic activation of T cells in the absence of an ongoing threat limits the ability of the immune system to respond effectively to new challenges. Supporting this, murine studies demonstrate impaired viral clearance and antigen-specific T cell responses following burn injury. Influenza-challenged mice four weeks after burn (8% TBSA, full-thickness) exhibit elevated viral titres in bronchoalveolar lavage fluid and lung tissue compared to non-burn mice [117]. Similarly, in a herpes simplex virus infection model, non-severe burn injury impairs CD8^+^ T cell expansion, effector function, and the expression of memory-associated markers [118]. Importantly, this study involved the adoptive transfer of transgenic CD8^+^ T cells (gBT.1) from non-burned donors into burn-injured mice, providing evidence that these defects in adaptive immune responses are driven by the post-burn microenvironment rather than intrinsic T cell dysfunction.

While the role of B cells in burn-associated immune dysfunction is less well defined, burn injury has been associated with reduced antibody responses to vaccination [8]. In this study, a substantial proportion of burn survivors fell below the protective threshold for sero-positivity for DTaP (diphtheria, tetanus, pertussis) antigens, despite confirmed post-burn vaccination. In parallel, targeted metabolomic profiling revealed distinct metabolic signatures in burn survivors, with clear differences between vaccine responders and non-responders [9]. Additionally, correlative analysis identified strong associations between circulating metabolites and cytokine levels exclusively in burn survivors, further strengthening the link between systemic metabolic alterations and impaired adaptive immune responses. For a significant proportion of patients, burn injury induces a sustained imbalance in adaptive immune regulation, characterised by persistent T cell activation alongside impaired functional and memory responses. This dysregulation persists well beyond clinical wound healing and contributes to long-term susceptibility to infection and impaired immune resilience. Importantly, both experimental and clinical evidence suggest that these immune alterations are closely linked to systemic metabolic changes, highlighting immunometabolism as a central regulatory axis in post-burn pathology. Several therapies currently used in burn care already modulate metabolic pathways and have emerging effects on adaptive immune function, suggesting potential opportunities for therapeutic repurposing to restore immune homeostasis following burn injury.

### 5.1. Strategy 1: Modulation of Stress Hormone Signalling via β-Adrenergic Blockade

Burn injury is associated with a persistent increase in stress hormones, including cortisol and catecholamines such as epinephrine and norepinephrine, which drives the hypermetabolic state observed following severe burns [55]. Accordingly, β-adrenergic blockade, most commonly using the non-selective β_1_/β_2_ antagonist propranolol, is widely used to attenuate catecholamine-driven hypermetabolism [119]. In paediatric patients with severe burns, propranolol has been shown to safely reduce muscle wasting, decrease cardiac workload, and improve metabolic outcomes, including hepatic lipid metabolism (reviewed in [120]). While randomised controlled trials in adults remain limited, retrospective analyses have shown β-blocker use is associated with reduced mortality after severe burn injury [119].

Beyond its metabolic effects, β-adrenergic signalling plays a direct role in regulating adaptive immune function. Norepinephrine, which remains significantly elevated following burn injury, signals through the β_2_-adrenergic receptor (β_2_-AR) expressed on T cells, activating intracellular pathways involving cyclic AMP and protein kinase A (reviewed in [121]). This signalling axis has been shown to suppress T cell activation, proliferation, and cytokine production, in part through effects on cellular metabolism and mitochondrial function. In addition, β_2_-AR signalling has been linked to increased expression of immune inhibitory receptors such as PD-1, promoting T cell exhaustion and dysfunction (reviewed in [122]). This recently discovered interaction between norepinephrine and T cells highlights a potential mechanistic link between sustained nervous system activation and impaired adaptive immune responses following burn injury. While the role of neuroimmune interactions in burns remains underexplored, modulation of β-adrenergic signalling using propranolol represents a promising strategy to limit chronic T cell dysfunction and restore immune balance with an affordable and relatively safe drug that is already routinely utilised in burn care.

### 5.2. Strategy 2: Modulation of Sex Hormones and Endocrine Homeostasis

Alongside excessive production of stress hormones, burn injury has been shown to significantly affect sex hormones, characterised by decreased testosterone, dysregulated estrogen signalling, and increased inflammatory mediators including prostaglandin E2 (reviewed in [6]). These changes, which have been found to persist for years after injury, contribute to ongoing hypermetabolism and immune dysregulation in affected patients. After burn injury, female sex is associated with poorer outcomes, including increased mortality and long-term morbidity [123,124,125]. Consistent with this, recent work demonstrates sex-specific differences in circulating metabolomic and lipid profiles after non-severe paediatric burns [126], suggesting that altered immunometabolic pathways may drive divergent immune responses between males and females. Together, these findings highlight the importance of understanding the role of sex-specific endocrine–immune interactions in post-burn recovery.

Therapeutically, modulation of androgen signalling represents an established approach to counteract burn-induced hypermetabolism. Oxandrolone, a synthetic testosterone analogue, is widely used in burn care, particularly in paediatric patients with severe burns where it effectively reduces muscle wasting and length of hospital stay [127]. While its primary effects are metabolic, androgen receptor signalling is increasingly recognised to influence immune cell function, in part through regulation of pathways such as master regulator of T cell regulation (mTOR) that are critical for T cell activation and differentiation [128]. Combination approaches may further enhance therapeutic efficacy; oxandrolone and propranolol have been shown to act synergistically to attenuate hypermetabolism, with evidence suggesting convergence on protein synthesis pathways, including mTOR, particularly when combined with exercise and nutritional support (reviewed in [129]). Despite these benefits, the direct effects of oxandrolone on adaptive immune responses remain poorly defined. Furthermore, variability in patient response, particularly across age groups, together with the risk of adverse effects such as hepatotoxicity in adults, highlights the need for improved patient stratification and further research into the immune-modulating mechanisms of this drug [129,130].

In contrast to testosterone, the role of estrogen in burn recovery remains poorly understood, despite clear sex disparities in outcomes. Estrogen exerts complex, dose-dependent effects on immune function and tissue repair, but emerging evidence suggests potential benefits of estrogen therapy following burn injury in specific populations, such as post-menopausal women [131]. However, significant heterogeneity in hormonal responses after burn injury, together with a lack of targeted clinical studies, currently limits therapeutic application. Advancing this area will require improved approaches to detect endocrine dysregulation and stratify patients, enabling more tailored interventions to address the disproportionate burden of poor outcomes observed in female burn patients.

### 5.3. Strategy 3: Targeting Immunometabolic Regulatory Pathways

Given the central role of metabolic dysregulation in burn-induced immune dysfunction, directly targeting immunometabolic pathways represents a promising therapeutic strategy. Current clinical practice for severe burns often already incorporates metabolic control strategies, most notably insulin therapy, which is used to manage burn-induced hyperglycaemia and insulin resistance, which commonly occurs after injury [132]. Beyond glycaemic regulation, insulin has been shown to exert immunomodulatory effects, including reducing systemic inflammation and influencing immune cell function [133,134], highlighting the broader link between metabolic control and immune outcomes in burn patients. Despite its benefits, insulin therapy is associated with risks, including risk of hypoglycaemia, warranting investigation of more targeted approaches.

Modulation of mTOR signalling, a key regulator of cellular metabolism, protein synthesis, and T cell differentiation, is emerging as a potential avenue to restore immune balance following injury. Metformin, a widely used anti-hyperglycaemic agent, has recently gained renewed attention for its immunomodulatory properties [135]. Mechanistically, metformin acts in part through activation of AMP-activated protein kinase (AMPK), leading to downstream inhibition of mTOR signalling. In immune cells, this shift promotes metabolic reprogramming, favouring oxidative metabolism over glycolysis, influencing T cell differentiation, enhancing memory T cell formation and modulating inflammatory responses. In the context of burn injury, where persistent hypermetabolism and immune dysregulation coexist, both insulin and metformin show the potential of metabolic interventions to modulate immune outcomes. While insulin primarily addresses acute metabolic dysfunction, metformin may offer complementary effects through longer-term immunometabolic reprogramming (reviewed in [135]). Although not currently part of standard burn care, a randomised controlled Phase II clinical trial in adults with severe burns demonstrated that metformin is safe and comparable in efficacy to insulin, without causing hypoglycaemia [136].

Outside of burn injury, metformin has recently been shown to improve vaccine responses in older adults by reversing aspects of T cell exhaustion and enhancing antigen-specific memory responses [137], suggesting a broader role in improving immunological resilience. Key questions to address for implementation in burn treatment include optimal timing of administration, patient selection, and how it interacts with existing therapies such as propranolol and oxandrolone. However, given its well-established safety profile and low cost, metformin represents a compelling candidate for repurposing in this setting. Future studies integrating metabolic, hormonal, and immune profiling will be critical to determine whether immunometabolic therapies can be effectively incorporated into personalised treatment strategies to restore immune function and improve outcomes following burn injury.

## 6. Emerging Strategies and the Future of Burn Care: Challenges and Considerations

Despite advances in acute burn care, immune dysfunction remains a significant and under-appreciated consequence of burn injury, contributing to the global health burden and reducing quality of life for burn patients. Burn injury triggers rapid and excessive systemic responses across inflammatory, endocrine, metabolic, and immune regulatory pathways. When left unresolved, this can lead to chronic immune dysfunction characterised by aberrant cytokine signalling, dysfunctional innate responses, and persistent activation and exhaustion of adaptive immune cells. This immune dysfunction reflects the complex consequences of PIICS, where both suppressive and pro-inflammatory processes compete and perpetuate each other [4]. Adding to this complexity, a growing appreciation of the essential role of the immune system in burns, not only in preventing infection but in directing the wound healing process, makes targeting this therapeutically particularly challenging, and current therapeutic strategies remain largely focused on managing acute symptoms rather than restoring immune homeostasis. Future advances in burn care should aim to move beyond symptom management towards targeted restoration of immune balance, which will improve both short- and long-term outcomes following burn injury.

### 6.1. Clinical Trajectories and Diagnostics

A major challenge in managing burn-induced immune dysfunction is the lack of standardized tools to define and monitor distinct clinical trajectories. Current markers, including white blood cell counts, C-reactive protein, procalcitonin, and composite severity scores, provide only a limited snapshot of immune status. A proposed addition to these parameters may be the use of the neutrophil-lymphocyte ratio [138], circulating cell-free DNA [139], immune phenotyping using flow cytometry or cytokine profiling [7], and metabolomic or lipidomic analyses to identify patients at risk of persistent dysregulation [109]. Early prognostic stratification of clinical trajectories and longitudinal monitoring, both during acute care and after healing, will be essential to detect immune alterations and implement appropriate interventions.

### 6.2. Towards Personalized Medicine

Burn patients represent a highly heterogeneous population as factors such as age, sex, burn size and depth, metabolic status, and pre-existing comorbidities all influence immune responses and recovery trajectories [140]. For example, young children and elderly patients exhibit distinct immune and metabolic profiles, which may necessitate tailored therapeutic strategies and dosing regimens [141]. Moving towards personalized medicine will require integration of clinical, immunological, and metabolic data to guide patient stratification and optimize treatment selection and dosing. Timing of therapy is critical, as the post-burn immune response is highly dynamic, with immune cell populations and inflammatory mediator levels fluctuating over time. Improved stratification of patients based on these parameters, alongside the identification of robust biomarkers of immune function across different stages of burn recovery, will be critical for advancing personalised treatment strategies. Furthermore, modulation of the microbiome represents an emerging and potentially valuable avenue to enhance immune resilience and improve clinical outcomes in burn patients [142].

### 6.3. Balancing Inflammation and Host Defence

A central challenge in burn care is achieving the appropriate balance between suppressing excessive inflammation and preserving essential antimicrobial defence. While hyperinflammation contributes to tissue damage and systemic complications, excessive immunosuppression increases susceptibility to infection and sepsis [3]. This balance is further complicated by the temporal dynamics of the immune response, as early inflammation is critical for pathogen clearance and wound healing, whereas later phases require resolution and restoration of immune competence. Therapeutic interventions must therefore be precisely timed and calibrated to modulate, rather than ablate, immune function.

### 6.4. Expanding Therapeutic Strategies

Beyond conventional pharmacological approaches, several underexplored strategies may contribute to improved outcomes. Interventions targeting immunometabolism, including agents such as propranolol or metformin, have been demonstrated to indirectly modulate immune responses by attenuating hypermetabolism [122,135,143]. In addition, nutritional support and structured exercise or rehabilitation programs have demonstrated benefits in severe burn populations and may hold promise for broader application, including in patients with non-severe burns. Drug repurposing from related inflammatory conditions, such as sepsis or COVID-19, also represents a valuable avenue.

Addressing burn-associated immune dysfunction will require a holistic and adaptive approach that integrates early source control, such as timely removal of burn eschar, with targeted immunomodulatory strategies. Combination therapies that act at multiple levels of the inflammatory cascade, together with improved longitudinal monitoring, may offer the greatest potential to restore immune homeostasis. The finding that pharmaceutical interventions already utilised in patients after severe burns have the potential to reduce or prevent immune dysfunction represent an attractive place to start, especially with common drugs like metformin, which are already widely used, relatively safe, easy to administer and low-cost, which is a final but important consideration given the high burden of burn injury in middle- and low-income countries or in situations like war. Ultimately, aligning personalized treatment strategies with detailed tracking of clinical and immunological trajectories will be key to improving long-term outcomes for burn patients.

## 7. Conclusions

Burn injury induces a complex and sustained immune dysregulation driven by interconnected inflammatory, metabolic, and endocrine pathways, which extends far beyond the acute phase of wound healing. While current therapies primarily address immediate complications, emerging evidence highlights the potential of targeted and repurposed interventions to timely restore immune homeostasis. Key therapeutic directions include early source control of inflammation through infection management and timely removal of eschar tissue; modulation of innate immune reactions to limit excessive neutrophil and macrophage-driven damage while preserving host defence; and targeting immunometabolic and endocrine pathways, for example via β-adrenergic blockade and metabolic agents such as metformin. In addition, repurposed and locally delivered immunomodulatory strategies may allow more precise control of inflammatory signalling. Future progress will depend on improved patient stratification, longitudinal immune monitoring, and a better understanding of temporal immune dynamics after burn injury. Ultimately, holistic approaches and personalized therapeutic strategies hold promise for improving outcomes in burn patients.

## Figures and Tables

**Table 1:** Common non-sterile triggers (PAMPs) and sterile triggers (DAMPs) for inflammation present during burn injury.

	Structure	Source	Receptor/Pathway
**Non-sterile triggers (PAMPs)**	Lipopolysaccharide (LPS)	Gram-negative bacteria	TLR4
	Peptidoglycan, lipoteichoic acid (LTA)	Gram-positive bacteria	TLR2 and NLRs
	Flagellin	Motile bacteria	TLR5
	Foreign carbohydrates	Fungi, bacteria, viruses	CLRs
	Foreign DNA/RNA	Viruses, bacteria	TLR3,7-9, RLRs, ALRs
**Sterile triggers (DAMPs)**	High-mobility group box-1 (HMGB1)	Nuclei of necrotic cells	TLR2,4 and RAGE
	S100 proteins	Cytoplasm of damaged cells	TLR4 and RAGE
	Heat shock proteins (HSP60,70)	Stressed/damaged cells	TLR2,4
	Endogenous glycans	Necrotic cells	CLRs
	Host DNA/RNA	Damaged cells	TLR3,7,9
	ATP	Cytosol of dying cells	P2X7 receptor of NLRP3 inflammasome; NLRs
	Substance P	Damaged nerves	NK1R
	Uric acid	Dying cells	NLRP3 inflammasome
	ROS (O_2_^−^, H_2_O_2_, OH molecules)	Oxidative burst	Keap1-Nrf2, NLRs, MAPKs, NF-κB
	NOS (iNOS, RNS, ONOO^−^, NO)	Oxidative burst	NLRP3, redox-sensitive kinases

Abbreviations: TLR = Toll-like receptor; NLR = NOD-like receptor; CLR = C-type lectin receptor; RAGE = Receptor for Advanced Glycation End Products; RLR = RIG-I-like receptor; ALR = AIM2-like receptor; NK1R = neurokinin 1 receptor.

## Data Availability

No new data were created or analyzed in this study. Data sharing is not applicable to this article.

## Abbreviations

The following abbreviations are used in this manuscript:

SIRS	Systemic inflammatory response syndrome
CARS	Compensatory anti-inflammatory response syndrome
PICS	Persistent inflammation immunosuppression and catabolism syndrome
PAMPs	Pathogen-associated molecular patterns
LPS	Lipopolysaccharide
LTA	Lipoteichoic acid
DAMPs	Damage-associated molecular patterns
TLR	Toll-like receptor
NLR	NOD-like receptor
CLR	C-type lectin receptor
RAGE	Receptor for advanced glycation end products
RLR	RIG-I-like receptor
ALR	AIM2-like receptor
NK1R	Neurokinin 1 receptor
PRRs	Pattern recognition receptors
HMBG1	High mobility group box 1
ETs	Extracellular traps
sRAGE	Soluble RAGE
TBSA	Total body surface area
U	Unknown (not investigated)
IL-	Interleukin
IFN-γ	Interferon gamma
TNF-α	Tumour-necrosis factor alpha
CXCL_	Chemokine C-X-C motif ligand
MCP-	Monocyte chemo-attractant protein
MIP-	Monocyte inducible protein
RANTES	Regulated on activation, normal T cell expressed and secreted
GROα	Growth-regulated oncogene alpha
CCL_	CC chemokine ligands
IP-10	Interferon-gamma-inducible protein 10 kDa
CTACK	Cutaneous T-cell-attracting chemokine
VEGF-A	Vascular endothelial growth factor
TGF-	Transforming growth factor
PDGF-	Platelet-derived growth factor
G-CSF	Granulocyte colony-stimulating factor
GM-CSF	Granulocyte-macrophage colony-stimulating factor
EGF	Epidermal growth factor
FGF	Fibroblast growth factor
HIF-1α	Hypoxia-inducible factor 1α
NF-κB	Nuclear factor kappa-light-chain-enhancer of activated B cells
SDF-1	Stromal cell-derived factor 1
GCs	Glucocorticosteroids
ROS	Reactive oxygen species
NET	Neutrophil extracellular trap
MPO	Myeloperoxidase
PAD4	Peptidylarginine deiminase 4
DCs	Dendritic cells
NK	Natural killer cells
Tregs	T regulatory cells
DTaP	Diptheria, tetanus, pertussis
mTOR	Master regulator of T cell regulation
AMPK	AMP-activated protein kinase
